# Observed to expected or logistic regression to identify hospitals with high or low 30-day mortality?

**DOI:** 10.1371/journal.pone.0195248

**Published:** 2018-04-13

**Authors:** Doris Tove Kristoffersen, Jon Helgeland, Jocelyne Clench-Aas, Petter Laake, Marit B. Veierød

**Affiliations:** 1 Division for Health Services, Norwegian Institute of Public Health, Oslo, Norway; 2 Division for Physical and Mental Health, Norwegian Institute of Public Health, Oslo, Norway; 3 Oslo Centre for Biostatistics and Epidemiology, Department of Biostatistics, Institute of Basic Medical Sciences, University of Oslo, Oslo, Norway; National Yang-Ming University, TAIWAN

## Abstract

**Introduction:**

A common quality indicator for monitoring and comparing hospitals is based on death within 30 days of admission. An important use is to determine whether a hospital has higher or lower mortality than other hospitals. Thus, the ability to identify such outliers correctly is essential. Two approaches for detection are: 1) calculating the ratio of observed to expected number of deaths (OE) per hospital and 2) including all hospitals in a logistic regression (LR) comparing each hospital to a form of average over all hospitals. The aim of this study was to compare OE and LR with respect to correctly identifying 30-day mortality outliers. Modifications of the methods, i.e., variance corrected approach of OE (OE-Faris), bias corrected LR (LR-Firth), and trimmed mean variants of LR and LR-Firth were also studied.

**Materials and methods:**

To study the properties of OE and LR and their variants, we performed a simulation study by generating patient data from hospitals with known outlier status (low mortality, high mortality, non-outlier). Data from simulated scenarios with varying number of hospitals, hospital volume, and mortality outlier status, were analysed by the different methods and compared by level of significance (ability to falsely claim an outlier) and power (ability to reveal an outlier). Moreover, administrative data for patients with acute myocardial infarction (AMI), stroke, and hip fracture from Norwegian hospitals for 2012–2014 were analysed.

**Results:**

None of the methods achieved the nominal (test) level of significance for both low and high mortality outliers. For low mortality outliers, the levels of significance were increased four- to fivefold for OE and OE-Faris. For high mortality outliers, OE and OE-Faris, LR 25% trimmed and LR-Firth 10% and 25% trimmed maintained approximately the nominal level. The methods agreed with respect to outlier status for 94.1% of the AMI hospitals, 98.0% of the stroke, and 97.8% of the hip fracture hospitals.

**Conclusion:**

We recommend, on the balance, LR-Firth 10% or 25% trimmed for detection of both low and high mortality outliers.

## Introduction

Mortality is commonly used as a quality indicator for monitoring and comparing hospitals, and is publicly reported in many countries [1, 2]. Death for any reason within 30 days of admission, occurring in the hospital or after discharge is frequently used for the calculation of the indicator. An important use is to determine whether a hospital has a significantly higher or lower mortality than other hospitals. Thus, the ability to identify such outliers correctly is essential [3]. Failure to identify a high mortality outlier hospital may severely influence patient safety, whereas incorrect status as a low-mortality hospital may lead to undeserved pay for performance or unwarranted lack of attention to quality.

During the past couple of decades, much of the literature has been concerned with variables that should be included to adjust for differences between hospital patient populations (case-mix) and statistical modelling techniques [4–15]. However, less is reported about the ability to falsely claim a hospital as an outlier (level of significance) and to reveal an outlier (power). One simple approach for outlier detection is to calculate the ratio of observed to expected number of deaths (OE) for each hospital. OE is based on the implicit assumption of negligible variability of the expected number of deaths. As this may cause bias in the variance estimate, a correction of the estimator has been proposed by Faris et al. [16]. Another approach for outlier detection is to include all hospitals in a logistic regression analysis (LR), comparing each hospital to a form of average over all hospitals [4]. A natural choice of average is the mean hospital mortality, after correction for case-mix, on the logistic scale. In our experience, some hospitals have very low mortality and will heavily distort this measure. One approach would then be to exclude such hospitals, but it is desirable to have a method that avoids exclusion of hospitals. One would then have to formulate objective criteria for such exclusions. Thus, a trimmed mean is more appropriate [17, 18]. In practice, hospitals with no deaths may be observed, in which case the maximum likelihood estimator for LR does not exist. To alleviate bias and convergence problems associated with zero or low numbers of deaths in some hospitals, Firth’s bias correction method for a logistic model can be used [19].

The aim of the present work was 1) to compare OE and LR, with respect to identifying outlier hospitals, 2) investigate whether modified versions of LR and OR improve the outlier detection. We compared the level of significance, power, and probability of directional error (claiming a high mortality hospital to be a low mortality hospital or vice versa) of the methods in a simulation study. In addition, the methods were compared by analysing patient data from all Norwegian hospitals for 2012–2014 for three medical conditions: first-time acute myocardial infarction (AMI), stroke and hip fracture. The motivation for this study was to investigate statistical properties of the two methods for using 30-day mortality as a quality indicator for all Norwegian hospitals, i.e., about 55 hospitals.

## Material and methods

To study the properties of OE and LR and their variants, we performed a simulation study by generating patient data from hospitals with known outlier status.

### Simulation study design

The simulation scenarios were designed to compare the methods with different combinations of number of hospitals, number of patients per hospital (volume), and levels of hospital mortality. We compared N = 10, 20, 50 or 100 hospitals, at a time. For each choice of N there were three categories for hospital volume: large (500), medium (300), and small (60). Ten different scenarios (A-J) were generated, and within each scenario true outlier status for each hospital was defined as low mortality outlier, non-outlier or high mortality outlier, Table 1. In the A scenarios all hospitals were non-outliers. A proportion of the large hospitals were high mortality outliers in the B scenarios, a proportion of the medium volume hospitals were high mortality outliers in the C scenarios, and a proportion of the small hospitals were high mortality outliers in the D scenarios. The scenarios E-J comprised different combinations of outlier status and hospital volume, Table 1.

**Table 1:** Design of simulation scenarios: Number of hospitals according to hospital volume (number of patients) and outlier status (low mortality, non-outlier, high mortality).

Hospital volume, number of patients per hospital volume category	Large, n = 500			Medium, n = 300			Small, n = 60		
**N = 10**	**2**			**3**			**5**		
**True outlier status**	**Low**	**Non-outlier**	**High**	**Low**	**Non-outlier**	**High**	**Low**	**Non-outlier**	**High**
**Scenario A**	0	2	0	0	3	0	0	5	0
**B**	0	1	1	0	3	0	0	5	0
**C**	0	2	0	0	2	1	0	5	0
**D**	0	2	0	0	3	0	0	3	2
**E**	1	0	1	0	3	0	0	5	0
**F**	1	0	1	1	1	1	0	5	0
**G**	1	0	1	0	3	0	1	3	1
**H**	0	1	1	0	3	0	1	3	1
**I**	0	1	1	0	2	1	3	1	1
**J**	0	2	0	0	3	0	0	4	1
**N = 20**	**3**			**6**			**11**		
**True outlier status**	**Low**	**Non-outlier**	**High**	**Low**	**Non-outlier**	**High**	**Low**	**Non-outlier**	**High**
**Scenario A**	0	3	0	0	6	0	0	11	0
**B**	0	2	1	0	6	0	0	11	0
**C**	0	3	0	0	4	2	0	11	0
**D**	0	3	0	0	6	0	0	6	5
**E**	1	1	1	0	6	0	0	11	0
**F**	1	1	1	1	4	1	0	11	0
**G**	1	1	1	0	6	0	1	9	1
**H**	0	2	1	0	6	0	1	9	1
**I**	0	2	1	0	5	1	2	9	0
**J**	0	2	1	0	5	1	0	10	1
**N = 50**	**5**			**14**			**31**		
**True outlier status**	**Low**	**Non-outlier**	**High**	**Low**	**Non-outlier**	**High**	**Low**	**Non-outlier**	**High**
**Scenario A**	0	5	0	0	14	0	0	31	0
**B**	0	3	2	0	14	0	0	31	0
**C**	0	5	0	0	8	6	0	31	0
**D**	0	5	0	0	14	0	0	18	13
**E**	1	3	1	0	14	0	0	31	0
**F**	1	3	1	2	10	2	0	31	0
**G**	1	3	1	0	14	0	1	29	1
**H**	0	4	1	0	14	0	1	29	2
**I**	0	4	1	0	13	1	2	29	0
**J**	0	4	1	0	13	1	0	30	1
**N = 100**	**10**			**35**			**55**		
**True outlier status**	**Low**	**Non-outlier**	**High**	**Low**	**Non-outlier**	**High**	**Low**	**Non-outlier**	**High**
**Scenario A**	0	10	0	0	35	0	0	55	0
**B**	0	2	1	0	35	0	0	55	0
**C**	0	10	0	0	18	17	0	55	0
**D**	0	10	0	0	35	0	0	35	20
**E**	1	8	1	0	35	0	0	55	0
**F**	1	8	1	2	31	2	0	55	0
**G**	1	8	1	0	35	0	2	51	2
**H**	0	9	1	0	35	0	2	51	2
**I**	0	9	1	0	34	1	2	51	2
**J**	0	9	1	0	34	1	0	53	2

### Observed to expected ratio (OE)

The probability of death within 30 days can be estimated using a logistic regression model.

Let *p*_*ij*_ denotel the probability of death for patient *j* at hospital *i*, *i* = 1, 2, …, *N* and j = 1, 2, …, *n*_*i*_ where *N* is the total number of hospitals and *n*_*i*_ is the number of patients at hospital *i*. *p*_*ij*_ is then given by the logistic model

$$\text{ln}\frac{p_{ij}}{1-p_{ij}}=\alpha +\gamma \prime z_{ij}$$ (1)

where *α* is a constant term, **z**_*ij*_ is the vector of case-mix variables for patient *j* at hospital *i*, and **γ** is the vector of regression coefficients. The parameters in (1) are estimated by the maximum likelihood method by using data from all hospitals or from a reference patient group, which gives parameter estimates $\hat{\alpha }$ and $\hat{\gamma }$, and inserted into (1) we find the estimated probabilities of death ${\hat{p}}_{ij}$.

For hospital *i*, the estimated, case-mix adjusted expected number of deaths is ${\hat{E}}_{i}=\sum_{j=1}^{n_{i}}{\hat{p}}_{ij}$ and *O*_*i*_ is the observed number of deaths. The ratio $O_{i}/{\hat{E}}_{i}$ can be used as a quality indicator for hospital *i* [4, 20]. A hospital with a ratio statistically significant below (above) one is a low (high) mortality outlier. If we assume the variance of the estimated expected number of deaths to be negligible, the estimated standard error of $O_{i}/{\hat{E}}_{i}$ is given by

$${\hat{SE}}_{OE_{i}}=\frac{1}{{\hat{E}}_{i}}\sqrt{\sum_{j=1}^{n_{i}}{\hat{p}}_{ij}{\left(1-{\hat{p}}_{ij}\right)}^{2}}.$$ (2)

The corresponding outlier test statistic is

$$Z_{OE_{i}}=\frac{O_{i}/{\hat{E}}_{i}-1}{{\hat{SE}}_{OE_{i}}}.$$ (3)

We denote the outlier detection method based on the test statistic (3) by OE. In practice, hospital volumes will often vary widely, in which case the parameters of the mortality model (1) will be strongly influenced by the largest hospitals.

The validity of the assumption of negligible variation in ${\hat{E}}_{i}$ for OE has been examined [16]. Faris et al. used propagation of error to derive a bias correction of the estimator, given by the expression (formula A.15 (22)) for the asymptotic standard error

$${\hat{SE}}_{OE-Faris}{}_{{}_{i}}=\frac{1}{{\hat{E}}_{i}}\sqrt{\{[(O_{i}/{\hat{E}}_{i}{)}^{2}-2O_{i}/{\hat{E}}_{i}]\hat{\text{Var}}(E_{i})+\hat{\text{Var}}(O_{i})\}}.$$ (4)

Note that ${\hat{SE}}_{OE_{i}}\geq {\hat{SE}}_{OE-Faris}{}_{{}_{i}}$ unless $O_{i}/{\hat{E}}_{i}\geq 2$.

We denote the outlier detection method using (4) for the standard error in the calculation of the test statistic for $O_{i}/{\hat{E}}_{i}$, by OE-Faris.

### Logistic regression (LR)

For direct comparison of the hospitals, the logistic regression model (1) is extended by including the hospital specific mortality parameters *μ*_*i*_, *i* = 1, …, *N*. The probability of death for patient *j* at hospital *i*, *p*_*ij*_, is now assumed to follow the logistic regression model

$$\text{ln}\frac{p_{ij}}{1-p_{ij}}=\mu_{i}+\gamma \prime z_{ij}$$ (5)

with **z**_*ij*_ and **γ** as in (1). In the standard model, the parameters of interest are the hospital effects, defined as the deviation from the mean of the *μ*_*i*_s (4)

$$\beta_{i}=\mu_{i}-\frac{1}{h}\sum_{i=1}^{h}\mu_{i}.$$ (6)

The maximum likelihood estimate ${\hat{\beta }}_{i}$ can be used as a quality indicator for hospital *i*: ${\hat{\beta }}_{i}$ is positive if hospital *i* performs worse (has higher mortality) than the average and vice versa. To test if hospital *i* is an outlier, i.e., significantly deviating from the mean, the test statistic $Z_{{\text{LR}}_{i}}={\hat{\beta }}_{i}/SE\left({\hat{\beta }}_{i}\right)$ is used. We denote this method by LR.

When the distribution of hospitals is heavy tailed, i.e., some hospitals have very low or very high mortality, the standard error of the sample mean can be relatively large and the mean is then a non-robust measure of location for the hospital effects [18, 21]. It is well known that trimmed means are more robust for heavy-tailed distributions, and are commonly used in such situations [17, 18]. Thus, as a location measure for the hospital effects we studied the mean and the trimmed mean (LR trimmed). For LR 5%, 10%, and 25% trimmed, the modified hospital effects *β*_*i*_ are defined as the deviation of the *μ*_*i*_ from their 5%, 10%, and 25% trimmed mean, respectively. The estimates of the modified effects are calculated from the standard maximum likelihood estimators by subtracting the trimmed means. The test statistic is $Z_{{\text{LR}}_{i}}$, where the variance of the mean-standardized effects is used as an approximation to the variance of the trimmed-mean-standardized effects.

For hospitals with no deaths, the maximum likelihood estimator does not exist. Asymptotic bias of the maximum likelihood estimator can be removed by a modification using a maximum penalized likelihood estimate, as shown by Firth [19]. We denote the resulting method by LR-Firth and the trimmed variants LR-Firth 5%, 10%, and 25% trimmed. Another approach, commonly used in practice to deal with hospitals with no deaths, is to omit them from the analysis. Thus, we also compared LR and LR-Firth when excluding 0-death hospitals.

### Simulation of data

To avoid ambiguity about true outlier status, we simplified the data generating process by letting hospitals of same outlier category have the same hospital parameter *μ*_*i*_. We used three different sets for *μ*_*i*_: one set corresponding to approximately 6.0% mortality for non-outlier hospitals (low mortality ≈3.9%, high mortality ≈9.0%), one set corresponding to approximately 9.8% for non-outlier hospitals (low mortality ≈7.5%, high mortality ≈12.8%), and one set corresponding to approximately 16.4% for non-outliers (low mortality ≈12.8%, high mortality ≈20.6%), Table 2. The *μ*_*i*_s for outliers were chosen to avoid very difficult (power close to nominal level) or very easy outlier identification (power close to 1).

**Table 2:** Sampling probabilities and input regression estimates for simulation scenarios, logistic scale. *μ*_*low*_, *μ*_*non*−*outlier*_, and *μ*_*high*_ are the hospital specific mortality effects for low mortality outliers, non-outliers, and high mortality outliers. *γ*_*sex*_ and *γ*_*age*_ are the regression coefficients for sex and age, respectively.

		Outlier status		
		Low, *μ*_*low*_ (average mortality)	Non-outlier, *μ*_*non*−*outlier*_ (average mortality)	High, *μ*_*high*_ (average mortality)
**Mortality, logistic scale**	**Set 1: Non-outliers have low mortality**	-7.0 (≈3.9%)	-6.55 (≈6.0%)	-6.1(≈9.0%)
	**Set 2: Non-outliers have medium mortality**	-6.3 (≈7.5%)	-6.0 (≈ 9.8%)	-5.7 (≈12.8%)
	**Set 3: Non-outliers have high mortality**	-5.7 (≈12.8%)	-5.4 (≈16.4%)	-5.11(≈20.6%)
**Case-mix, logistic scale**	Sex ~ Bernoulli(1, 0.4), *γ*_*sex*_ = -3			
	Age ~ Beta(7.5, 2.5, scale = 100), *γ*_*age*_ = 0.05			

Two case-mix variables were included: sex and age. Sex was sampled from a Bernoulli distribution with 40% females. Age was generated from a beta distribution with parameters *a* = 7.5 and *b* = 2.5 for all scenarios and scaled by 100, Table 1. Our choices correspond roughly to the age distribution and proportion of females for patients with AMI in Norway (see Results).

Data were simulated from the logistic model (5) by generating 10,000 replications for each scenario according to Table 1 by using parameter estimates as given in Table 2.

#### Hospital administrative patient data

The Norwegian Patient Registry receives hospital administrative patient data from all Norwegian hospitals, and provided the following data: type of admission (acute or elective), coded medical diagnoses and medical procedures, date and time for admission/discharge, age, and gender. Records for AMI, stroke, and hip fracture were identified according to ICD-10 [22] [22]: first time AMI (I21.0–3,9), stroke (I61, I63, I64), and hip fracture (S72.0–2). Date of death was retrieved from the National Registry. Unique personal identification numbers for all Norwegian residents enabled linkage of data sources; for details see [23]. Deaths from any cause, occurring in- or out-of-hospital within 30 days of hospital admission were used for the estimation of 30-day mortality. We included the first episode of care for each patient with AMI, stroke, or hip fracture during 2012–2014. For patients treated at two or more hospitals during one episode of care, the episode was assigned to the first hospital. All hospitals with 60 or more admissions per medical condition during the 3-year period were included: 51 hospitals for AMI, 51 for stroke, and 45 hospitals for hip fracture. For the purpose of this study, we included only sex and age as covariates. The data for each medical condition were analysed by the different methods, using 0.05 nominal level of significance for outlier testing.

The Norwegian Data Inspectorate and the Ministry of Health approved the data collection. Because the project employs only existing administrative data for quality improvement purposes, approval from the Regional Ethical Committee was not required.

### Comparison of the methods

For all the above methods, a hospital is declared a low (high) mortality outlier, at nominal *ε* level of significance, if the test statistic is smaller (larger) than the lower (upper) *ε*-percentile of the standard normal distribution. Otherwise, a hospital is categorized as a non-outlier.

#### Simulated data

For each iteration of the 10 000 runs, the data generated was analysed by OE and LR and their variants, assigning low mortality outlier status, non-outlier, and high mortality outlier status according to 0.01, 0.02, 0.05, and 0.10 nominal level of significance. For each method, scenario and mortality parameter set, actual level of significance was estimated as the proportion of non-outlier hospitals classified as low mortality outlier and as high mortality outlier. Power was estimated as the proportion of hospitals correctly classified as low or high mortality outlier. Directional error probability was estimated as the proportion of outliers classified as outliers in the wrong direction, i.e., low mortality hospitals that were identified as high mortality outliers or vice versa.

For each method, the scenarios were summarized by maximum actual level of significance (estimate for level of significance), and the mean power (estimate for power), as well as maximum directional error probability.

#### Hospital data

The hospital data did not contain any 0-death hospitals. The data for each medical condition were analysed by the ten different methods: OE, OE-Faris, LR and LR 5%, 10% and 25% trimmed, and LR-Firth, LR-Firth 5%, 10% and 25% trimmed. The hospitals were counted according to concurring status and the number of hospitals for which their status was altered from non-outlier to high/low outlier by a subset of the methods. Fleiss’ kappa was calculated for assessing agreement of outliers across the methods. Strength of agreement was evaluated according to the Kappa cut-offs given by Landis and Koch [24]: <0.00 = ‘Poor’, 0.00–0.20 = ‘Slight’, 0.21–0.40 = ‘Fair’, 0.41–0.60 = ‘Moderate’, 0.61–0.80 = ‘Substantial’, 0.81–1.00 = ‘Almost Perfect’.

The simulations and analyses were performed in R [25].

## Results

### Simulation study

**Fig 1:**
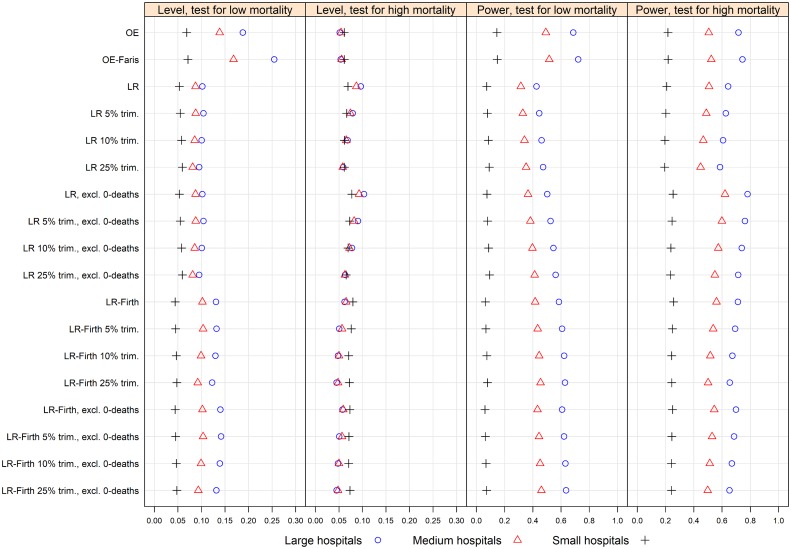
Results of the simulation study. Level of significance and power for the different methods for one-sided tests at 0.05 nominal level per hospital volume and outlier category, aggregated over all scenarios A-J, number of hospitals compared, and the three mortality sets. OE = the ratio of observed to expected number of deaths; OE-Faris = variance corrected OE; LR = logistic regression using maximum likelihood; LR-Firth = LR with bias correction; LR 5%, 10% and 25% trim. = trimmed mean variants of LR; LR-Firth 5%, 10%, and 25% trim. = trimmed mean variants of LR-Firth; excl. 0-deaths = excluding hospitals with no deaths.

None of the methods achieved nominal level of significance for all hospital volume categories, when testing for both low and high mortality outliers. Trimming improved the actual level of significance for LR and LR-Firth, but decreased the power. Aggregated over all scenarios A-J, number and volume of hospitals compared, and the three mortality parameter sets, Fig 1 shows the actual level of significance and power for one-sided tests at 0.05 nominal level per hospital volume and outlier category.

Plots and tables of summary statistics according to number of hospitals compared and hospital mortality set are available in Supporting Information files S1–S3 Figs and S1 and S2 Tables.

#### Level of significance

When testing for low mortality outliers at 0.05 nominal level of significance, Fig 1 panel 1, OE and OE-Faris showed the highest actual level of significance, ranging from around 0.06 for small hospitals to around 0.19 (OE) and 0.25 (OE-Faris) for large hospitals. The high error probabilities were typically found for asymmetric cases, with large or medium hospitals being high mortality outliers (data not shown). The actual levels for LR and variants varied less, ranging from slightly above 0.05 for small hospitals, to slightly above 0.10 for large hospitals. For LR-Firth and variants, nominal level of significance was achieved for small hospitals, and the actual level was below 0.15 for medium and large hospitals. The high error probabilities for LR-Firth and variants typically occurred for asymmetric cases where small hospitals were high mortality outliers (data not shown).

When testing for high mortality outliers, the variation in actual level of significance was less: around 0.10 and lower across hospital volume categories, Fig 1 panel 2. LR-Firth 10% and 25% trimmed variants achieved nominal level of significance for large and medium hospitals. For small hospitals, actual level of significance was around 0.07 for LR-Firth and LR-Firth trimmed variants. Actual level of significance ranged from 0.05 to 0.065 for all hospital volumes for OE, OE-Faris, and LR 10% and 25% trimmed variants. When excluding hospitals with zero deaths, LR had the highest actual level of significance.

#### Power

When testing for low mortality outliers at 0.05 nominal level, the power ranged from below 0.1 for small hospitals to around 0.7 for large hospitals, Fig 1 panel 3. LR and LR trimmed variants had lowest power (below 0.6 for all hospital volumes). OE and OE-Faris had somewhat higher power than the other methods. When testing for high mortality hospitals, the power was higher and ranged from about 0.2 for small hospitals to 0.8 for large hospitals, for all methods, Fig 1 panel 4. Trimming improved the actual level of significance for LR and LR-Firth, but decreased the power.

#### Directional error probability

At 0.05 nominal level, maximum directional error was below 0.016 for all methods and scenarios considered (results included in Supporting information S1 and S2 Tables). Results for tests at nominal 0.01 level were similar to those for 0.05 level (data not shown).

### Patient data

Hospital and patient characteristics are summarized in Table 3.

**Table 3:** Hospital and patient characteristics, data from Norwegian hospitals 2012–2014.

	AMI	Stroke	Hip fracture
**Number of hospitals, (number of patients)**	51 (33 950)	51 (26 935)	45 (24 258)
**Number of patients per hospital, median (range)**	463 (103–3794)	416 (78–2261)	452 (69–1838)
**Overall unadjusted 30-day mortality (hospital range)**	11.3% (8.0%–20.4%)	13.4% (8.4%–21.5%)	8.9% (4.8%–12.1%)
**Age, years, mean (standard deviation)**	71.6 (14.1)	74.6 (13.6)	83.4 (8.0)
**Females (%)**	37.9%	47.3%	71.1%

The methods agreed with respect to outlier status for 48 out of 51 AMI hospitals, Table 4. Of the remaining AMI hospitals, two were identified as high mortality outliers by LR 10% and 25% trimmed and LR-Firth 10% and 25% trimmed. One additional AMI hospital was identified as a high mortality outlier by LR-Firth 25% trimmed. For stroke, the methods agreed for 50 out of 51 hospitals. The remaining hospital was identified as a high mortality outlier by OE and OE-Faris. For hip fracture, the methods agreed for 44 out of 45 hospitals. The remaining hospitals was identified as low mortality hospital by LR-Firth, LR-Firth 25% trimmed, LR 25% trimmed, OE, and OE-Faris. No hospital changed status from low mortality to high mortality or vice versa. All Fleiss’ kappa values above 0.94 and indicated almost perfect agreement, Table 4.

**Table 4:** Number of hospitals and status (low mortality, non-outlier, high mortality) according to the various methods; the ratio of observed to expected number of deaths (OE), variance corrected OE (OE-Faris), logistic regression (LR), bias corrected LR (LR-Firth), trimmed mean variants (LR trimmed and LR-Firth trimmed). Fleiss’ kappa for agreement across methods.

		AMI	Stroke	Hip fracture
**Hospital status, identified by all methods**	**Low**	2	9	3
	**Non-outlier**	40	38	38
	**High**	6	3	3
**High mortality according to**	**LR 10% trimmedLR 25% trimmedLR-Firth 10% trimmedLR-Firth 25% trimmed**	2	0	0
	**LR-Firth 25% trimmed**	1	0	0
	**OEOE-Faris**	0	1	0
**Low mortality according to**	**LR-FirthLR-Firth 25% trimmedLR 25% trimmedOEOE-Faris**	0	0	1
**Fleiss’ kappa**		0.94	0.99	0.96

## Discussion

We compared the level of significance, power, and probability of directional error for OE and LR, with and without modifications in a simulation study. None of the methods were superior overall with respect to both level of significance and power for detection of both low and high mortality outlier hospitals.

The various methods for estimating 30-day mortality and profiling hospitals include e.g. empirical Bayesian methods, hierarchical/multilevel models, and regression trees [4–6, 8, 12]. Comparisons of methods with respect to estimation or hospital outlier detection have been done for selected medical conditions and by simulations [26–28]. Multilevel methods have been reported to be more conservative than methods based on fixed effects [5, 11][9] and to have convergence problems [9]. Whether to use a Baysian or a frequentist approach is still debated [29]. However, it is generally accepted that for the purpose of testing hypotheses about individual hospitals this should be formulated in a fixed effects model [30–32]. As our purpose was to make inferences about the Norwegian hospitals, we used fixed effect models. In practice, the testing is followed by estimating the mortality for each hospital. For this purpose, it is common practice to shrink the estimated regression coefficients for each hospital towards the location measure by a hierarchical Bayesian method [6, 7]. This is also done for estimating mortality (survival) for Norwegian hospitals [23].

Standard theory only guarantees control over asymptotic error probabilities under the exact null hypotheses *β*_*i*_ = 0 for the hospital in question for the LR-methods (and similarly for OE). One can easily visualize situations where this is not the same as outlier status, e.g. the scenarios used in the simulation experiment. Thus, we may expect levels of significance different from the nominal level. In terms of having actual level of significance close to the nominal level for all three hospital volume categories, OE and OE-Faris were best when testing for high mortality outliers, and LR performed worst. However, nominal level was achieved by LR-Firth 10% and 25% trimmed for large and medium volume hospitals. When testing for low mortality outliers, the actual level of significance increased considerably, particularly for OE and OE-Faris. The most plausible explanation is the so-called swamping effect: When multiple outliers are present, they distort the distribution of the presumed non-outliers, which is the basis for comparison when assessing outlier status of a single observation [33][18]. When using OE, the estimated expected number of deaths under the null hypothesis of equal probabilities of deaths across hospitals is weighted by the number of cases in each hospital. In practice, hospital volumes will often vary widely, in which case the parameters of the mortality model (1) will be strongly influenced by the largest hospitals. OE is thus subject to a hospital volume effect which we regard as a weakness of the OE approach. In the standardizing condition of the LR model, however, the hospital (case-mix adjusted) average is unweighted, because the objective is to measure the performance of each hospital relative to the hospital population. Thus, the LR hospital effects can be regarded as hospital number-weighted measures of the probability of death. OE is appropriate for outlier detection but not for inter-hospital comparisons because the case-mix adjustment is based on the risk factor distribution of the hospital under evaluation rather than a common distribution. The question arises of whether a different weighting scheme could be introduced in OE (and OE-Faris). The main appeal of OE is its simplicity, which would be lost if a different scheme was used. Alternatively, hospital volume, as measured by the number of cases, could be used for weighting in the LR methods. We would then have measured the performance relative to the patient population. A hospital can with minimal analytic expertise, calculate its OE when parameter estimates are provided. For LR, all data from all hospitals need to be available and thus more expertise, usually an institution outside the hospitals, performs the calculations. The advantage of the latter, more complex approach is that hospitals can be compared directly versus the location measure and versus other hospitals.

With respect to power when testing for low mortality outliers, OE, OE-Faris, and LR-Firth and trimmed variants performed best, and LR worst. In practice, hospitals with no deaths are not included when compared by LR and trimmed variants. When excluding such 0-deaths hospitals, LR and trimmed variants perform well for power when testing for high mortality, as visualized by using the low mortality outlier set. For the simulations scenarios with the low mortality set, there were few replications where deaths occurred in all hospitals. Thus, the convergence of the iterative LR method was uncertain, making the estimates for actual level of significance and power unreliable [34].

The simulation scenarios showed very low power for small hospitals. The Centres for Medicare & Medicaid Services found that ≥500 patients per hospital was appropriate for identifying poor performers [35]. This is concordant with our findings. Many Norwegian hospitals are small, and despite using data for a 3-year period, requiring at least 20 cases per year [23], the simulations suggest that we may miss small, outlying hospitals, independent of method. One can argue that when testing small hospitals, a somewhat increased level of significance may be acceptable, if accompanied by higher power. However, due to the excess level of significance for OE and OE-Faris when testing for low mortality outliers among medium and large hospitals, only LR-Firth and trimmed variants and LR excluding 0-deaths 10% and 25% trimmed showed overall more reliable performance. Somewhat surprisingly, OE-Faris did not improve on OE. The reason may be that we compared 10 or more hospitals in the simulations.

Our simulation study covered several scenarios for number of hospitals and ways of allocation of outlier status to hospitals. The variation of hospital volume was chosen to mimic the actual distribution in Norway, and presumably representative of the situation elsewhere. Thus, we believe the probability model is realistic for 30-day mortality. In addition, we tested the methods on real data for three medical conditions. The simulation scenarios were designed so that true outlier status was unambiguous. This might have been more realistic by having a continuous distribution of mortality among the hospitals. However, one would then have to make a definition of outlier status in terms of the true parameter values. Our comparisons could then be contingent on the choice of definition, which is not evident. Future studies could investigate this problem. We have not covered scenarios where the case-mix, e.g. age distribution, differs between hospitals. In principle, we could have included hierarchical or multilevel methods in the comparison. However, as already noted, these methods have been found to be inferior for the outlier detection problem. In practice, one would use correction for multiple testing. The most common methods rely on component tests for individual hospitals [36, 37], so that correction for multiplicity would not invalidate our conclusions.

When using hospital data, the objective was to compare the methods by analysing the same data to see whether the identification of outliers differed with the different methods. We could have used the last episode of care, or randomly selected an episode. For the sake of simplicity, we chose the first episode of care for a patient. The methods agreed with respect to outlier status for 94.1% of the AMI hospitals, 98.0% of the stroke and 97.8% of the hip fracture hospitals. However, LR-Firth 25% trimmed identified the largest number of outliers, three high mortality and one low mortality outliers. The simulation data showed good performance for LR 25% trimmed, LR-Firth, and LR-Firth 10% and 25% trimmed. The results for the hospital data were in agreement with this.

### Summing up

One should carefully consider the choice of method for outlier detection, and be aware that the actual level of significance may be higher than the nominal. In particular, none of the methods we considered had satisfactory level when testing for low mortality outliers. A possible solution is to use different levels for the two kinds of test, e.g. 0.05 when testing for high mortality outliers, and 0.02 when testing for low mortality outliers. Another would be to use resampling methods, e.g. bootstrapping, to ensure correct level of significance.

In our opinion, 30-day mortality is as an important indicator for guiding quality improvement. In particular, hospitals identified as outliers may have suboptimal care and need to evaluate whether their practice is in accordance with guidelines, and take action to improve quality [38]. Falsely claiming a hospital to be a low mortality hospital may give a hospital a misleading well-performing reputation and undeserved economic advantages. Falsely claiming a high mortality outlier can do harm by leading to closing down of departments or no renewal of contracts [39]. When weighing the risk of not identifying a high mortality hospital versus the risk of false classification of a hospital to have low mortality, we are of the opinion that detecting high mortality hospitals is more important than identifying low mortality hospitals. For a single method applicable to detection of both high and low mortality outliers, we recommend, on the balance, LR-Firth 10% or 25% trimmed.

## Supporting information

S1 Fig Results of the simulation study using low mortality set for hospital outlier status, actual level of significance and power across scenarios A-J. N = number of hospitals compared; OE = the ratio of observed to expected number of deaths; OE-Faris = variance corrected OE; LR = logistic regression; LR-Firth = LR with bias correction; LR 5%, 10% and 25% trim. = trimmed mean variants of LR; LR-Firth 5%, 10%, and 25% trim. = trimmed mean variants of LR-Firth; excl. 0-deaths = excluding hospitals with no deaths.
(7Z) Click here for additional data file.

S2 Fig Results of the simulation study using medium mortality set for hospital outlier status, actual level of significance and power across scenarios A-J. N = number of hospitals compared; OE = the ratio of observed to expected number of deaths; OE-Faris = variance corrected OE; LR = logistic regression; LR-Firth = LR with bias correction; LR 5%, 10% and 25% trim. = trimmed mean variants of LR; LR-Firth 5%, 10%, and 25% trim. = trimmed mean variants of LR-Firth; excl. 0-deaths = excluding hospitals with no deaths.
(7Z) Click here for additional data file.

S3 Fig Results of the simulation study using high mortality set for hospital outlier status, actual level of significance and power across scenarios A-J. N = number of hospitals compared; OE = the ratio of observed to expected number of deaths; OE-Faris = variance corrected OE; LR = logistic regression; LR-Firth = LR with bias correction; LR 5%, 10% and 25% trim. = trimmed mean variants of LR; LR-Firth 5%, 10%, and 25% trim. = trimmed mean variants of LR-Firth; excl. 0-deaths = excluding hospitals with no deaths.
(7Z) Click here for additional data file.

S1 Table Results from simulation study when testing for low mortality outlier hospitals: Mean and range for actual level of significance, power, and directional error; per method for the three mortality sets and number of hospitals compared, across scenarios A-J per hospital volume. (XLSX)

S2 Table Results from simulation study when testing for high mortality outlier hospitals: Mean and range for actual level of significance, power, and directional error; per method for the three mortality sets and number of hospitals compared, across scenarios A-J per hospital volume. (XLSX)

S1 Dataset Aggregated data pr simulation scenario for each category of hospital volume and hospital mortality set according to each method of analysis. Number of hospitals for Z-values of 0%, 0.1%, 0.2%, 0.5%, 1.0%, 2.0%, 5.0%, 10.0%, 90.0%, 95.0%, 98.0%, 99.0%, 99.5%, 99.9%, 100.0%.
(CSV)

S2 Dataset Results from one iteration of each simulation scenario using low mortality set for the hospitals. (CSV)

S3 Dataset Results from one iteration of each simulation scenario using medium mortality set for the hospitals. (CSV)

S4 Dataset Results from one iteration of each simulation scenario using high mortality set for the hospitals. (CSV)
